# Whole-Genome Sequences of Two NDM-1-Producing Pseudomonas aeruginosa Strains Isolated in a Clinical Setting in Albania in 2018

**DOI:** 10.1128/MRA.01291-19

**Published:** 2020-01-02

**Authors:** Silva Tafaj, Floriana Gona, Célia F. Rodrigues, Perlat Kapisyzi, Fatmir Caushi, John W. Rossen, Daniela M. Cirillo

**Affiliations:** a Microbiology Department, University Hospital Shefqet Ndroqi, Tirana, Albania; b Emerging Bacterial Pathogens Unit, Division of Immunology, Transplantation, and Infectious Diseases, IRCCS San Raffaele Scientific Institute, Milan, Italy; c LEPABE, Department of Chemical Engineering, Faculty of Engineering, University of Porto, Porto, Portugal; d Pneumology Department, University Hospital Shefqet Ndroqi, Tirana, Albania; e Thoracic Surgery Department, University Hospital Shefqet Ndroqi, Tirana, Albania; f Department of Medical Microbiology and Infection Prevention, University of Groningen, University Medical Center Groningen, Groningen, The Netherlands; University of Rochester School of Medicine and Dentistry

## Abstract

Isolation of metallo-β-lactamase-producing, carbapenem-resistant, Pseudomonas aeruginosa strains is increasingly being documented worldwide; their presence constitutes a public health threat. Here, we report draft genome sequences of two New Delhi metallo-β-lactamase-1-producing, multidrug-resistant, P. aeruginosa strains of sequence type 235 that were isolated from the surgical wound of two patients hospitalized in the same ward.

## ANNOUNCEMENT

Pseudomonas aeruginosa isolates belonging to sequence type 235 (ST235), an international high-risk clone that has the potential to cause nosocomial outbreaks with poor clinical outcomes, are a cause of serious concern. A recent study (1) estimated that the ST235 sublineage emerged in Europe around 1984 and has successfully spread worldwide since then. Antibiotic inactivation through metallo-β-lactamase (MBL) possession is one of the resistance mechanisms. New Delhi MBL-1 (NDM-1)-producing P. aeruginosa strains have been reported in Serbia, Romania, (2, 3), and Italy (4) but not in Albania. The presence of this enzyme in Albania was first documented in 2018 in a Klebsiella pneumoniae isolate from a digestive carrier (5). Little is known regarding the spread of MBLs in Albania. A case of a K. pneumoniae carbapenemase 3 (KPC-3)-producing K. pneumoniae isolate was described in 2015 (6). Here, we report the genome sequences of two NDM-1-producing P. aeruginosa strains of ST235 (PA4 and PA5) that were isolated from the surgical wound of two patients hospitalized in the same ward.

Species identification was performed with the BBL Crystal enteric/nonfermenter identification kit (Becton, Dickinson, Sparks, MD), and results were confirmed by matrix-assisted laser desorption ionization–time of flight (MALDI–TOF) mass spectrometry on a MALDI Biotyper system (Bruker Daltonics, Germany).

Bacterial cultures were purified for DNA extraction by two successive single-colony selections after streaking on blood agar medium (Becton, Dickinson) and incubation overnight at 37°C. DNA was extracted from a liquid suspension of the purified cultures by using the Maxwell SEV 16-cell DNA purification kit, in combination with a Maxwell 16 instrument, to perform automated isolation of genomic DNA.

All strains were sequenced at the San Raffaele Hospital (Milan, Italy) on the NextSeq 500 platform (Illumina, Inc., San Diego, CA), with a paired-end run of 300 cycles, after Nextera XT library preparation, targeting a minimum coverage of 50-fold. Output raw reads were trimmed using Trimmomatic v.0.33 software to remove the adapters. Cleaned reads were used for *de novo* assembly with SPAdes v.3.6.1 (7) using the following parameters: PHRED quality offset for the input reads of 33, “careful mode” (which reduces the number of mismatches and short indels and also runs Mismatch Corrector, a postprocessing tool that uses the BWA tool), and default *k*-mer length settings to set *k*-mer lengths of 21, 33, 55, and 77. The quality of the assemblies was checked using a quality control tool for high-throughput sequence data, FastQC v.0.11.8 (https://www.bioinformatics.babraham.ac.uk/projects/download.html#fastqc).

The assembled contigs were evaluated with ResFinder v.3.0 (8), which is available from the Center for Genomic Epidemiology (http://www.genomicepidemiology.org), and Resistance Gene Identifier (RGI) v.5.1.0 from the Comprehensive Antibiotic Resistance Database (CARD), v.3.0.5 (9) (http://arpcard.mcmaster.ca). ResFinder was used for the specific identification of acquired resistance genes, while RGI was used to complement the data for resistome prediction, including not only acquired resistance but also intrinsic and mutation-driven resistance. The following parameters were used with RGI: selection of perfect and strict hits only, exclusion of the nudge of loose hits with ≥95% identity to strict hits, and high sequence quality and coverage. Multilocus sequence typing (MLST) was performed using the P. aeruginosa PubMLST database (10) (https://pubmlst.org/paeruginosa ). Core-genome MLST (cgMLST) and whole-genome MLST (wgMLST) were performed using SeqSphere+ v.5.1.1 (Ridom, Muenster, Germany).

The read length was 300 cycles, and the numbers of total reads for each strain were 2,323,831 for PA4 and 1,837,472 for PA5. The assembly of PA4 resulted in 480 contigs (*N*_50_, 37,820 bp) comprising 6,941,401 bp, with a GC content of 66.1%. The assembly of PA5 resulted in 507 contigs (*N*_50_, 37,045 bp) comprising 6,887,548 bp, with a GC content of 66.3%.

Through the CARD, a total of 58 antibiotic resistance genes were identified in PA4 (19 perfect hits and 39 strict hits), including genes conferring resistance to β-lactams, aminoglycosides, fluoroquinolones, macrolides, and tetracyclines through different mechanisms, such as antibiotic efflux (*n* = 37), antibiotic efflux and antibiotic target alteration (*n* = 3), antibiotic inactivation (*n* = 11), antibiotic target alteration (*n* = 6), and antibiotic target replacement (*n* = 1). PA5 expressed all 58 antibiotic resistance genes of PA4 plus the antibiotic efflux pump gene *mexY* (19 perfect hits and 40 strict hits). RGI results for PA4 and PA5 are summarized in Table 1.

**TABLE 1:** RGI results for PA4 and PA5

Strain	ARO^*a*^ term	RGI criteria
PA4	AAC(6')-Il	Perfect
	*adeF*	Strict
	ANT(2'')-Ia	Perfect
	APH(3')-IIb	Strict
	*arnA*	Strict
	*basR*	Strict
	*basS*	Strict
	*bcr-1*	Strict
	FosA	Strict
	MexA	Perfect
	MexB	Strict
	MexC	Strict
	MexD	Strict
	MexE	Strict
	MexF	Perfect
	MexG	Perfect
	MexH	Strict
	MexI	Strict
	MexJ	Strict
	MexK	Perfect
	MexL	Perfect
	*mexM*	Strict
	*mexN*	Strict
	*mexP*	Strict
	*mexQ*	Strict
	MexR	Strict
	MexS	Strict
	MexT	Strict
	MexV	Strict
	MexW	Strict
	MexZ	Strict
	MuxA	Strict
	MuxB	Perfect
	MuxC	Perfect
	*nalC*	Strict
	*nalD*	Strict
	NDM-1	Perfect
	OpmB	Perfect
	OpmD	Strict
	*opmE*	Strict
	OpmH	Perfect
	OprJ	Strict
	OprM	Perfect
	OprN	Strict
	OXA-488	Perfect
	PDC-2	Strict
	PmpM	Strict
	*Pseudomonas aeruginosa* catB7	Strict
	*Pseudomonas aeruginosa* CpxR	Perfect
	*Pseudomonas aeruginosa emrE*	Perfect
	*Pseudomonas aeruginosa gyrA* conferring resistance to fluoroquinolones	Strict
	*Pseudomonas aeruginosa soxR*	Perfect
	*qacH*	Strict
	*sul1*	Perfect
	TriA	Strict
	TriB	Perfect
	TriC	Strict
	Type A NfxB	Strict
PA5	AAC(6')-Il	Perfect
	*adeF*	Strict
	ANT(2'')-Ia	Perfect
	APH(3')-IIb	Strict
	*arnA*	Strict
	*basR*	Strict
	*basS*	Strict
	*bcr-1*	Strict
	FosA	Strict
	MexA	Perfect
	MexB	Strict
	MexC	Strict
	MexD	Strict
	MexE	Strict
	MexF	Perfect
	MexG	Perfect
	MexH	Strict
	MexI	Strict
	MexJ	Strict
	MexK	Perfect
	MexL	Perfect
	*mexM*	Strict
	*mexN*	Strict
	*mexP*	Strict
	*mexQ*	Strict
	MexR	Strict
	MexS	Strict
	MexT	Strict
	MexV	Strict
	MexW	Strict
	*mexY*	Strict
	MexZ	Strict
	MuxA	Strict
	MuxB	Perfect
	MuxC	Perfect
	*nalC*	Strict
	*nalD*	Strict
	NDM-1	Perfect
	OpmB	Perfect
	OpmD	Strict
	*opmE*	Strict
	OpmH	Perfect
	OprJ	Strict
	OprM	Perfect
	OprN	Strict
	OXA-488	Perfect
	PDC-2	Strict
	PmpM	Strict
	*Pseudomonas aeruginosa catB7*	Strict
	*Pseudomonas aeruginosa* CpxR	Perfect
	*Pseudomonas aeruginosa emrE*	Perfect
	*Pseudomonas aeruginosa gyrA* conferring resistance to fluoroquinolones	Strict
	*Pseudomonas aeruginosa soxR*	Perfect
	*qacH*	Strict
	*sul1*	Perfect
	TriA	Strict
	TriB	Perfect
	TriC	Strict
	Type A NfxB	Strict

a ARO, Antibiotic Resistance Ontology.

ResFinder identified genes responsible for acquired resistance to aminoglycosides [*aph(3ʹ)-IIb*, *ant(2ʺ)-Ia*, and *aac(6ʹ)-Il*], β-lactams (*bla*_PAO_, *bla*_NDM-1_, and *bla*_OXA-488_), fluoroquinolones (*crpP*), fosfomycin (*fosA*), phenicols (*catB7*), and sulfonamides (*sul1*). cgMLST showed 8 of 4,283 allele differences, whereas only 10 of 5,188 allele differences were found using wgMLST.

### Data availability.

The whole-genome shotgun project has been deposited in GenBank under BioProject accession number PRJNA522042. The BioSample accession numbers are SAMN10923322 for PA4 and SAMN10923323 for PA5.
